# Function Identification of the Nucleotides in Key *cis*-Element of *DYSFUNCTIONAL TAPETUM1* (*DYT1*) Promoter

**DOI:** 10.3389/fpls.2017.00153

**Published:** 2017-02-17

**Authors:** Shumin Zhou, Hongli Zhang, Ruisha Li, Qiang Hong, Yang Li, Qunfang Xia, Wei Zhang

**Affiliations:** Lab of Plant Development Biology, School of Life Sciences, Shanghai UniversityShanghai, China

**Keywords:** *Arabidopsis*, *DYT1*, *cis*-element, tissue specificity, tapetum, Through site mutation assay it was found that the “T” and final “C” nucleotides of key *cis*-element “CTCC” of *Arabidopsis* tapetum gene *DYT1* promoter were irreplaceable for tissue specific gene expression.

## Abstract

As a core regulatory gene of the anther development, *DYSFUNCTIONAL TAPETUM1* (*DYT1*) was expressed in tapetum preferentially. Previous study had confirmed that a “CTCC” sequence within *DYT1* promoter was indispensable for correct *DYT1* expression. However, precise analysis on the function of each nucleotide of this sequence still lacks. Here we employed site mutation assay to identify the function roles of the nucleotides. As a result, the “T” and final “C” of “CTCC” were found essential for the temporal and spatial specificity of *DYT1* expression, whereas the other two “C” nucleotides exhibited substitutable somewhat. The substitutes of two flanking nucleotides of “CTCC,” however, hardly affected the normal promoter function, suggesting that the “CTCC” sequence as a whole did meet the standard of a canonical *cis*-element by definition. In addition, it was found that as short as 497 bp *DYT1* promoter was sufficient for tissue-specific expression, while longer 505 bp *DYT1* promoter sequence was sufficient for species-specific expression.

## Introduction

Anther development is crucial for successful pollen production in flowering plants. The *Arabidopsis* anther during meiosis is a four-lobed structure comprised of concentric outer epidermis, endothecium, middle fibrous layer, tapetum and pollen mother cell (PMC; Goldberg et al., 1993; Yeung et al., 2011). The tapetum initially turns out as a single-cell layer surrounding PMC, and is the main nutrient tissue of PMC and pollen subsequently in the anther (Koltunow et al., 1990; Scott et al., 2004; Feng and Dickinson, 2007; Zhang et al., 2014; Li et al., 2015). A serial of regulatory genes have been identified to be essential for the tapetum function in *Arabidopsis* up to date, including *DYSFUNCTIONAL TAPETUM1* (*DYT1*), *DEFECTIVE IN TAPETAL DEVELOPMENT AND FUNCTION1* (*TDF1*), *MYB103/MYB80, ABORTED MICROSPORE* (*AMS*), *MALE STERILITY1* (*MS1*), etc. (Zhang et al., 2006, 2007; Yang et al., 2007; Zhu et al., 2008; Phan et al., 2011; Wang et al., 2012; Fernández-Gómez and Wilson, 2014; Xu et al., 2014, 2015; Shumin et al., 2015; Yi et al., 2016). Among them, *DYT1* as one of the earliest tapetum-preferential genes, initiates all aspects of tapetum function through regulating transcription of approximately 1,000 anther genes involved in callose synthesis and degradation, peptide and lipid transport, exine formation, etc. (Schiefthaler et al., 1999; Higginson et al., 2003; Sorensen et al., 2003; Ito et al., 2007; Liu et al., 2009; Feng et al., 2012; Phan et al., 2012; Li et al., 2013; Cui et al., 2016).

The expression profile of *DYT1* is highly tissue-specific. Weak expression of *DYT1* can be detected in the secondary parietal cell and sporogenous cell, the precursors of tapetum and PMC respectively at as early as anther stage 4 (Zhang et al., 2006; Shumin et al., 2015). Then *DYT1* expression significantly enhances and culminates with maturation of tapetum at the anther stages 6, and exhibits as a tapetum-preferential pattern (Zhang et al., 2006; Shumin et al., 2015). With the end of meiosis of PMC, *DYT1* expression declines rapidly, and disappears at stage 8 (Zhang et al., 2006; Gu et al., 2014; Shumin et al., 2015). The underlying mechanism(s) controlling *DYT1* temporal and spatial expression pattern remains as a puzzle since *DYT1* was firstly characterized one decade ago (Zhang et al., 2006). It has been known that at least two signal pathways are involved in initiation of *DYT1* expression. The first one seems to be governed by transcription regulatory factors, including nuclear proteins NZZ/SPL and LFR, and SBP-domain transcription factor SPL8 (Yang et al., 1999; Xing et al., 2010; Wang et al., 2012). The second pathway is mediated by protein phosphorylation triggered by a series of receptor-like kinases, such as EXS/EMS1, SERK1 and SERK2, BAM1 and BAM2 (Zhao et al., 2002, 2008; Albrecht et al., 2005; Colcombet et al., 2005; Hord et al., 2006; Li et al., 2017). Both signal pathways are essential for normal *DYT1* expression, though few details are known about how they crosstalk and activate *DYT1* expression together (Zhang et al., 2006; Shumin et al., 2015).

In our previous study, it had been confirmed that as short as 513 bp sequence in front of the transcription start site (TSS) of *DYT1* was essential and sufficient for proper temporal and spatial specificity of *DYT1* expression. In addition, the deletion of a “CTCC” sequence at the position of −468 bp (i.e., 468 bp from the TSS) abolished *DYT1* expression completely at the anther stage 6, suggesting that the “CTCC” sequence was indispensable for normal *DYT1* expression (Shumin et al., 2015). Including our previous study, there have been only a couple of related reports about “CTCC” as a putative *cis*-element crucial for gene expression regulation in plants (Kano-Murakami et al., 1991; Ku et al., 2011). However, whether the “CTCC” sequence is a canonical *cis*-element in which the nucleotides are irreplaceable, remains to be addressed. In this study, we employed site mutation assay to characterize the function roles of the nucleotides, including the two flanking ones of the “CTCC” sequence to answer the question whether the “CTCC” sequence met the standard of a canonical *cis*-element or not. In addition, more truncation analysis was performed through using both transgenic *Arabidopsis* and tobacco bright yellow 2 (BY2) cell suspensions to identify which regions of *DYT1* promoter were essential for tissue, and further species specificity of *DYT1* expression.

## Materials and methods

### Plant materials and growth conditions

*Arabidopsis thaliana* ecotype Col-0 was used in all of the transformation and promoter analysis in this study. The plants were cultivated under 16 h light/8 h dark photoperiod with 300 Es^−1^m^−2^ illumination intensity, at 22 ± 1°C. The seeds were stratified at 4°C for 4 days prior to growth.

The tobacco (*Nicotiana tabacum* L. cv Bright Yellow 2, BY2) was cultivated in a modified liquid Murashige and Skoog (MS) medium (Zhou et al., 2014) at 28°C with 120 rpm shaking avoiding light and maintained by weekly dilution (V/V = 1/10) of cell.

### Transformation constructs

**Figure 1:**
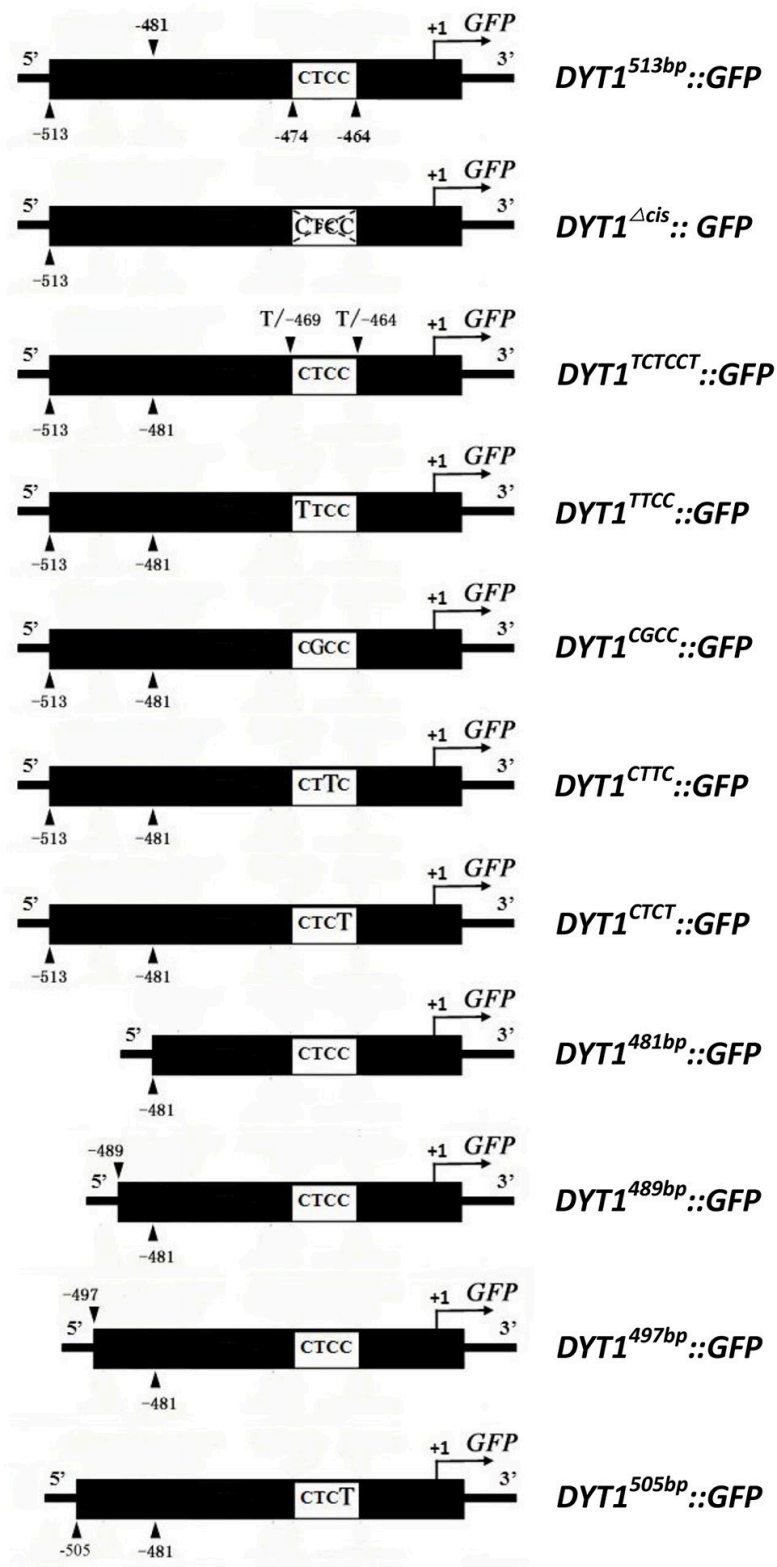
**Promoter constructs used to drive ***GFP*** expression in transgenic plant anthers and BY2 cells**.

The pre-existing 513 bp *DYT1* promoter-driven GFP expression construct, designated as *DYT1*^513*bp*^*::GFP* (Shumin et al., 2015), was used as PCR template in this study. The primers to generate site mutations of the constructs*DYT1*^*TTCC*^*::GFP, DYT1*^*CGCC*^*::GFP, DYT1*^*CTTC*^*::GFP* and *DYT1*^*CTCT*^*::GFP*; CTCC flanking site mutation construct*DYT1*^*TCTCCT*^*::GFP* were designed and synthesized respectively. Novel 5′ end primers of truncation constructs *DYT1*^489*bp*^*::GFP, DYT1*^497*bp*^*::GFP* and *DYT1*^505*bp*^*::GFP* were designed and synthesized, respectively (Figure 1). The PCR products were obtained and cloned into pCAMBIA1300 to make reporting constructs according to the report of Zhou (Shumin et al., 2015).

### Plant transformation

Transgenic plants were generated via floral-dip transformation. The positive transgenic seedlings were screened on MS medium containing 25 mg/L hygromycin (Clough and Bent, 1998). At least 10 independent transgenic T1 generation lines for each construct were observed in this study.

### BY2 cell suspension transformation

The transformation of BY2 suspension was carried out according to the report of Zhou (Zhou et al., 2014). BY2 cell suspension was co-cultivated with the *Agrobacterium* GV3101 strain harboring transgenic construct in liquid medium without antibiotics avoiding light at 28°C for 48 h, so that the final concentration of cell suspension was approximately OD_600_ = 0.6. The resulted BY2 cell suspension was enriched by centrifuge and plated on MS solid medium containing 50 μg/ml hygromycin and 100 μg /ml vancomycin, and incubated at 28°C avoiding light. Two weeks later, grown-up antibiotics-resistant callus were subjected to amplified liquid cultivation, and the resulted BY2 cell suspension was used for genotyping and fluorescence observation. At least 10 independent original antibiotics-resistant callus were observed for each construct. The pre-existing transgenic callus of cauliflower mosaic leaf virus 35S promoter-driven GFP expression *35S::GFP* was used as a positive control (Zhou et al., 2014).

### Semi-quantification PCR

Total RNA was extracted from the transgenic BY2 cell suspension and performed reverse transcription according to Zhou et al. (2014). Then *GFP* cDNA fragment was PCR amplified with *GFP* specific (*GFP* RT-F&R) primers with the sequence listed in Table 1.

**Table 1:** **PCR primers in this study**.

**Primer name**	**Sequence (5′–3′)**
p*DYT1F*-513	CCCAAAGCTTCTAACGTTGGACCTGTGGACT
p*DYT1*F-505	CCCAAAGCTTGGACCTGTGGACTCAGTTTAC
p*DYT1*F-497	CCCAAAGCTTTTTACAGAGCCGTGGTCGAGCCTC
p*DYT1*F-489	CCCAAAGCTTGGACTCAGTTTACAGAGCCGTGG
p*DYT1F*-481	CCCAAAGCTTGCCGTGGTCGAGCCTCCGC
p*513F*^Δcis^	CCCAAAGCTTTGGACCTGTGGACTCAGTTTACAGAGCCGTGGTCGAGCGCGAGGTG
p*513F^*TCTCCT*^*	CTAACGTTGGACCTGTGGACTCAGTTTACAGAGCCGTGGTCGAGTCTCCTCGAGGTGTGGAG
p*513F^*TTCC*^*	CTAACGTTGGACCTGTGGACTCAGTTTACAGAGCCGTGGTCGAGCTTCCGCGAGGTGTGGAG
p*513F^*CGCC*^*	CTAACGTTGGACCTGTGGACTCAGTTTACAGAGCCGTGGTCGAGCCGCCGCGAGGTGTGGAG
p*513F^*CTTC*^*	CTAACGTTGGACCTGTGGACTCAGTTTACAGAGCCGTGGTCGAGCCGCCGCGAGGTGTGGAG
p*513F^*CTCT*^*	CTAACGTTGGACCTGTGGACTCAGTTTACAGAGCCGTGGTCGAGCCTCTGCGAGGTGTGGAG
p*DYT1R*-co	CGGAGCTCTTATTTCTTCTTCTTTGATAATT
p*GFP*-RT-F	ATGGTGAGCAAGGGCGAGGAG
p*GFP*-RT-R	TTACTTGTACAGCTCGTCC
p*513^*d*1^*F	CCCAAAGCTTACAGAGCCGTGGTCGAGCGCGAG
p*513^*c*1^*F	CCCAAAGCTTACAGAGCCGTGGTCGAGCCTCC
p*513^*i*1^*F	TTACAGAGCCGTGGTCGAGTCTCCT
p*513^*i*2^*F	CAGTTTACAGAGCCGTGGTCGAGCT
p*513^*i*3^*F	AGTTTACAGAGCCGTGGTCGAGCCG
p*513^*i*4^*F	GTTTACAGAGCCGTGGTCGAGCCTT
p*513^*i*5^*F	TTTACAGAGCCGTGGTCGAGCCTCT
P*513R*-co	TTATTTCTTCTTCTTTGATAATT

### Observation of GFP fluorescence

Anthers were stripped and collected from transgenic plants flower bud just around male meiosis (anther stage 4–9) on a microscopy slides. Added one drop of sterile water on the anthers and covered a slide carefully without squeezing. Then the sample was observed and photographed under Zeiss LSM-710 confocal microscope (Zeiss, Germany) and Leica DM2500 fluorescence microscope. As for semi-quantification of the fluorescence intensity, randomly 10 sites on fluorescence images were selected and the intensity was measured and normalized by the SMART software. Statistics of at least 15 anthers per line, 10 independent T1 generation transgenic lines were counted for each construct transformation. As for BY2 cell suspension, at least 100 cells per callus ancestor were observed, and 10 callus were counted for each construct transformation.

## Results

### Two nucleotides of “CTCC” *cis*-element were essential for the accurate expression pattern of *DYT1* gene

**Figure 2:**
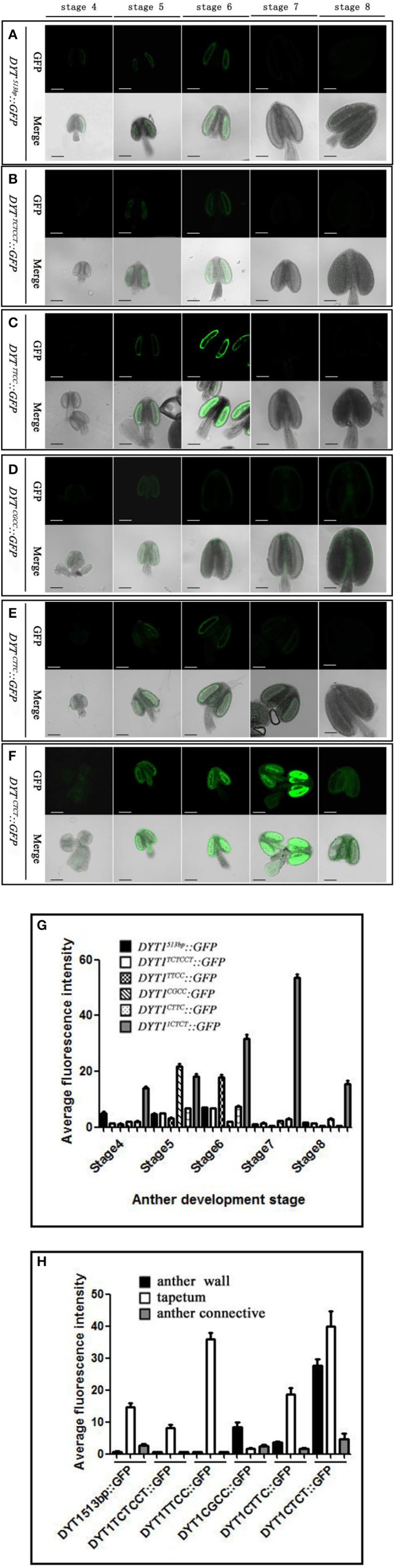
***GFP*** **expression in “CTCC” site mutation transgenic plant anthers. (A–F)** Green fluorescence images of anthers at stage 4–8 in *DYT1*^513*bp*^ *::GFP, DYT1*^*TCTCCT*^*::GFP, DYT1*^*TTCC*^*::GFP, DYT1*^*CGCC*^::*GFP, DYT1*^*CTTC*^::*GFP*, and *DYT1*^*CTCT*^ ::*GFP* in transgenic plants. **(G,H)** Semi-quantification of the average fluorescence intensity in stage 4–8 anthers and in different parts of stage 6 anthers of *DYT1*^513*bp*^*::GFP, DYT1*^*TCTCCT*^*::GFP, DYT1*^*TTCC*^*::GFP, DYT1*^*CGCC*^*::GFP, DYT1*^*CTTC*^*::GFP*, and *DYT1*^*CTCT*^ *::GFP* transgenic plantsthrough SMART software assay (*n* ≥ 30, ±*SD*; *p* < 0.1, Student's *t*-test), values were obtained from 3 independent lines of transgenic plants. Bar = 10 μm.

Previous studies showed that the 513 bp length *DYT1* promoter could faithfully regenerate the temporal and spatial profile of native *DYT1* expression (Shumin et al., 2015). The GFP signal of transgenic *DYT1*^513*bp*^*::GFP* firstly appeared in the secondary parietal cell and microsporocyte of stage 4 *Arabidopsis* anther. Then the GFP expression increased significantly and reached its peak preferentially in the tapetum of stage 5 and 6 anthers. Subsequently, the GFP signal rapidly weakened at stage 7 and disappeared at stage 8 (Figures 2A,G). The “CTCC” *cis*-element locating at −468 bp from the TSS is particularly important for the correct expression of the *DYT1* gene. The deletion of “CTCC” completely knocked out GFP expression (Shumin et al., 2015). To investigate the function of each nucleotide in the “CTCC” *cis*-element, a series of modifying constructs based on *DYT1*^513*bp*^*::GFP* with site mutations in or around the “CTCC” sequence were made, and transformed into *Arabidopsis*, respectively (Figure 1). The transgenic plants were identified by PCR using nucleotide specific primers (Table 1) and restriction endonuclease digestion assay (Supplementary Figure 1). The site mutations of the two flanking nucleotides of the “CTCC” *cis*-element (5′ end from “C” to “T,” and 3′ end from “C” to “T,” respectively), and the first and third nucleotide substitutes from “C” to “T” in the “CTCC” imposed no effect on the expression pattern of GFP (Figures 2B,C,E,G). On the contrast, however, “G” replacing “T” in the “CTCC” resulted in weak expression of *GFP* in the connective and epidermis tissues in addition to the tapetum and PMC (before stage 6, then microspore at stage 7 and 8; Figures 2D,H). Furthermore, “T” replacing the final “C” resulted in strong *GFP* expression in all tissues of stage 4–8 anthers (Figures 2F,H). Thus, the “T” and final “C” of the “CTCC” *cis*-element were suggested to play predominant roles in controlling the tissue specificity and appropriate intensity of the gene expression.

### As short as 497 bp *DYT1* promoter was sufficient for tissue-specific expression

**Figure 3:**
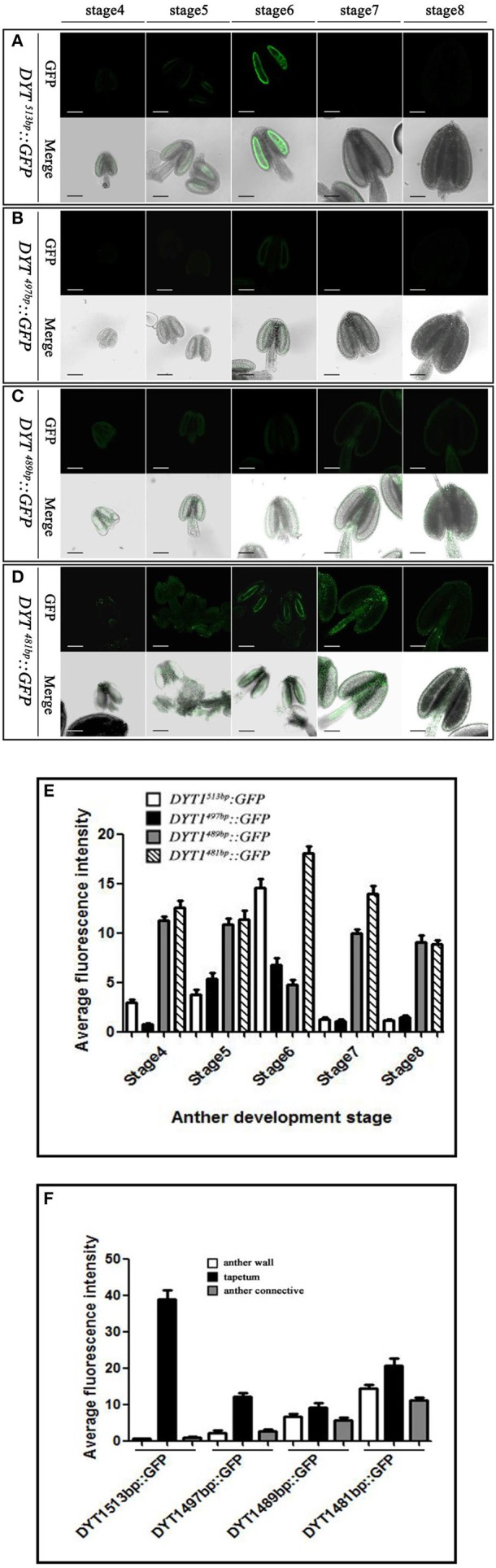
**GFP expressing in ***DYT1***^**513*****bp***^***::GFP***, ***DYT1***^**489*****bp***^***::GFP***, ***DYT1***^**497*****bp***^***::GFP and DYT1***^**481*****bp***^***::GFP*** transgenic Arabidopsis anthers. (A)** The green-fluorescence concentrates in the tapetal cells of transgenic plant anthers; **(B–D)** Obvious green-fluorescence displays both in the anther locules, tapetum and epidermis. The upper row are images of fluorescence, and the under row are merged images of light and fluorescence. **(E,F)** Semi-quantification of the average fluorescence intensity in stage4-8 anthers and in different parts of stage 6 anthers of *DYT1*^513*bp*^*::GFP, DYT1*^497*bp*^*::GFP, DYT1*^489*bp*^*::GFP*, and *DYT1*^481*bp*^*::GFP* transgenic plants through SMART software assay (*n* ≥ 30, ±*SD*; *p* < 0.1, Student's *t*-test), values were obtained from 3 to 5 independent lines of transgenic plants. Bar = 10 μm.

The previous study had elucidated that beside the core motif “CTCC,” the −481 to −513 bp region of *DYT1* promoter was also indispensable for appropriate expression. To uncover finer structure within this region, in addition to original 481 and 513 bp truncated *DYT1* promoter-driven *GFP* reporter constructs, 489, 497, and 505 bp truncated *DYT1* promoter-driven *GFP* reporter constructs were made and transformed into *Arabidopsis*, respectively. As a result, both 505 and 497 bp *DYT1* promoter gave rise of the identical expression pattern as the 513 bp *DYT1* promoter (Figures 3A,B,E), suggesting as short as 497 bp *DYT1* promoter was sufficient to recapitulate appropriate *DYT1* expression in *Arabidopsis* anther. On the other side, in *DYT*^489*bp*^*::GFP* transgenic plants, *GFP* exhibited an ectopic and weaker expression losing the tapetum-preferential pattern, similar to that of the 481 bp *DYT1* promoter. The detectable green fluorescence was distributed not only in the tapetum and PMC (before stage 6, then microspore at stage 7 and 8), but also in the connective and epidermis tissues (Figures 3C,D,F), suggesting that the sequence from −489 to −497 bp in *DYT1* promoter was essential for tapetum-preferential expressing pattern, and as short as 497 bp *DYT1* promoter sequence was sufficient for tissue-specific expression.

### 505 bp *DYT1* promoter was sufficient for species-specific expression

**Figure 4:**
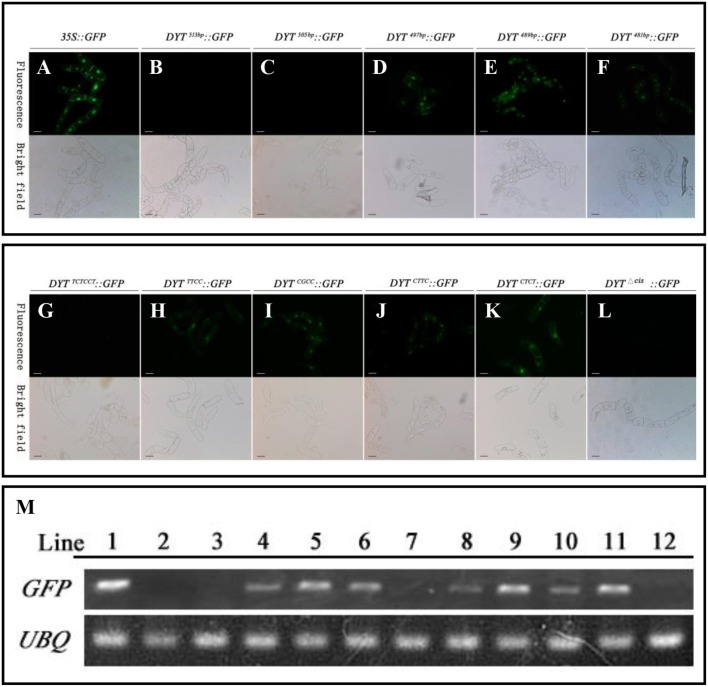
**Functional segments assay of ***DYT1*** promoterin transgenic BY2 cells**. GFP fluorescence was detected under Confocal microscope. **(A–F)** The fluorescence and bright images of GFP driven by *35S* promoter and different truncated *DYT1* promoters. **(G–L)** The function assay of “CTCC” segment in *DYT1* promoter driving GFP expression in BY2 cells. **(M)** RT-PCR analysis of *GFP/ACTIN* expression in different transgenic BY2 cells. Lane 1–12 represented *35S::GFP, DYT1*^513*bp*^*::GFP*,*DYT1*^505*bp*^*::GFP, DYT1*^497*bp*^*::GFP, DYT1*^489*bp*^*::GFP, DYT1*^481*bp*^::*GFP, DYT1*^*TCTCCT*^ ::*GFP, DYT1*^*TTCC*^::*GFP, DYT1*^*CGCC*^*::GFP, DYT1*^*CTTC*^*::GFP, DYT1*^*CTCT*^ *::GFP*, and *DYT1*^Δ^^*cis*^*::GFP* transgenic cells. Bar = 20 μm.

As mentioned before, the flanking −489 to −497 bp region seemed to play as a restriction element to limit *DYT1* expression with certain spaces so that *DYT1* expression exhibited as a specific spatial profile. Then one more question was brought up whether there was other region in 513 bp *DYT1* promoter imparting the species specificity. In order to test such possibility, the series of truncated *DYT1* promoter-driven *GFP* reporting constructs were transformed into tobacco BY2 cell suspension. In *DYT1*^497*bp*^*::GFP, DYT1*^489*bp*^*::GFP* and *DYT1*^481*bp*^*::GFP* transgenic BY2 cell suspension, weaker GFP expression comparing with that of *35S::GFP* transgenic cells was found (Figures 4A,D–F,M). However, in *DYT1*^513*bp*^*::GFP* and *DYT1*^505*bp*^*::GFP* transformed cell lines, no GFP signal could be detected (Figures 4B,C,M). Thus, 505 bp *DYT1* promoter sequence was sufficient for restricting the gene expression in *A. thaliana* rather than in other species such as tobacco BY2 cell suspension.

Furthermore, all site mutations within “CTCC” based on *DYT1*^513*bp*^*::GFP* gave rise of ectopic GFP expression in BY2 cell suspension, suggesting that the “CTCC” *cis*-element participated in determining species specificity. However, the substitutes of the “T” and final “C” generated stronger ectopic expression than the other two nucleotides (Figures 4I,K), suggesting the “T” and final “C” also contributed in determining species specificity more than the other two “C” nucleotides (Figures 4H,J), though not so exclusively as in determining tissue specificity in *Arabidopsis*. Consistent to the results obtained from *Arabidopsis* study (Figure 2), the mutations of “CTCC” flanking nucleotides had no effect on the driven gene expression (Figure 4G), further supporting “CTCC” itself was a four-nucleotides motif. Unlike site mutations, the “CTCC” deletion *DYT1*^513*bp*Δ*cis*^*:: GFP* generated little GFP fluorescence either in *Arabidopsis* anther (Shumin et al., 2015), or in BY2 cell suspension (Figures 4L,M), adding complexity to the function of intact “CTCC.” One explanation is that in addition to controlling spatial expression pattern of the driven gene, “CTCC” as a whole is also important for gene expression activation.

## Discussion

The findings of this study that the substitutes of the “T” and final “C” nucleotides in the “CTCC” sequence dramatically changed the driven gene (*GFP* here) expression profile, whereas the nucleotide replaces just out of “CTCC” imposed little effect on either tissue or species specificity, confirmed that the “CTCC” sequence did satisfy the definition of a canonical *cis*-element, and play as the core role in determining *DYT1* expression profile. Furthermore, with more “CTCC” flanking sequences truncated from *DYT1* promoter, the extent of gene expression specificity became weaker, reflected in the facts that the species specificity was lost firstly, then the expression region extended from the central locule to the connective tissue and epidermis in the *Arabidopsis* anther, resulting in a constitutive pattern at last. Thus it was suggested that *DYT1* promoter was a functional unit comprised of multiple parts whose absence would lead to expression specificity attenuation, from both species and tissue-specific to only tissue-specific, and finally to constitutive. In other words, the core motif “CTCC” and its flanking sequences need work together to restrict the driven gene expressed precisely in specific tissues, and furthermore in specific species.

With the key *cis*-element identified, undoubtedly the main task of next stage work is to identify the *trans*-factor(s) which recognizes and binds to the “CTCC” *cis*-element, and finally activates *DYT1* expression. As mentioned before, among the known *DYT1* upstream regulatory factors which are involved in transcription regulation, SPL8 participates in the small RNA signaling in cell differentiation regulation in anther. As a SBP domain factor, the DNA-binding motif of SPL8 is zinc-binding motif rather than “CTCC” (Xing et al., 2010). Furthermore, both SPL/NZZ and LFR lack functional DNA-binding domain (Yang et al., 1999; Wang et al., 2012). Thus, it is proposed that the regulatory factor recognizing and binding to the “CTCC” *cis*-element of *DYT1* promoter still needs to be characterized in future work. This unknown factor might be unable to activate *DYT1* expression alone. Conversely it would associate with SPL/NZZ and/or LFR to form an active transcription complex to trigger *DYT1* expression.

## Author contributions

WZ and SZ designed all experiments, analyzed data, and wrote the manuscript. HZ performed experiments on transgenic expression assays. RL worked on the transgenic lines. QH performed experiments on construction of transformation vector. YL performed analysis of promoter function element. QX performed statistical analysis of fluorescence intensity.

### Conflict of interest statement

The authors declare that the research was conducted in the absence of any commercial or financial relationships that could be construed as a potential conflict of interest.
