# Supplementary material for: Function Identification of the Nucleotides in Key cis-Element of DYSFUNCTIONAL TAPETUM1 (DYT1) Promoter
Source: Front Plant Sci. 2017 Feb 17;8:153. doi: 10.3389/fpls.2017.00153 (PMC5313476; doi:10.3389/fpls.2017.00153)
Supplement: Supplementary file 1 [file DataSheet1.docx]

**A**


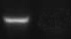


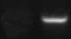


***DYT^513bp△cis^::GFP***

***DYT^513bp^::GFP***

**p*513^c1^F***

**p*513^d1^F***

**Supplementary figure 1. Genotype detection in the transgenic plants.**

Identification of genotype in transgenic plants, (A) the intact 513bp long promoter and ‘CTCC’ deleted promoter driving transgenic plants; (B) the intact 513bp long promoter and‘TCTCCT’promoterdriving transgenic plants;(C) the intact 513bp long promoter and‘TTCC’promoterdriving transgenic plants;(D) the intact 513bp long promoter and‘CGCC’promoterdriving transgenic plants; (E) the intact 513bp long promoter and‘CTTC’promoterdriving transgenic plants; (F) the intact 513bp long promoter and‘CTCT’promoterdriving transgenic plants; p*513*^c1^F, p*513*^i1^F, p*513*^i2^F, p*513*^i3^F, p*513*^i4^F and p*513*^i5^Fmeans the results of RT-PCR amplifying by identification primer as in Tab 1. (G)Enzymatic digestion results by Sac**Ⅰin** transgenic plants. **‘TR’ means the transgenic plants.**


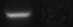


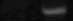


***DYT^CTCT^::GFP***

***DYT^513bp^::GFP***

**p*513^c1^F***

**p*513^i5^F***

**F**


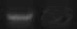

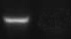


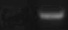


***DYT^TTCC^::GFP***

***DYT^513bp^::GFP***

**p*513^c1^F***

**p*513^i2^F***

**C**


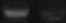


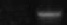


***DYT^CGCC^::GFP***

***DYT^513bp^::GFP***

**p*513^c1^F***

**p*513^i3^F***

**D**


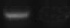


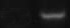


***DYT^CTTC^::GFP***

***DYT^513bp^::GFP***

**p*513^c1^F***

**p*513^i4^F***

**E**


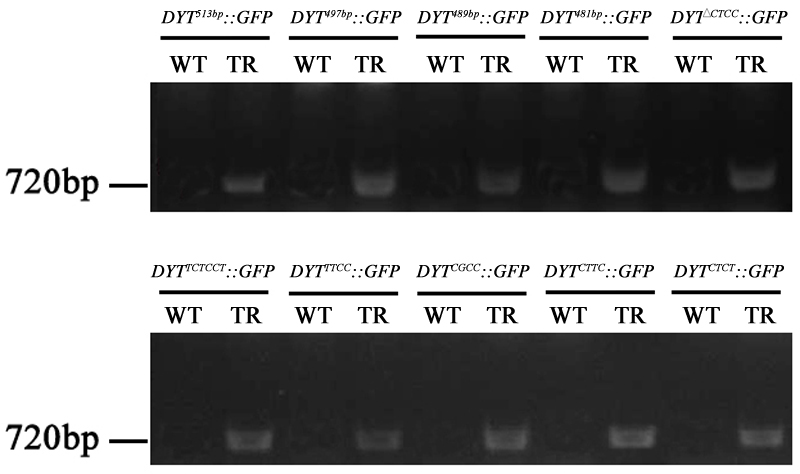


**G**
